# A conceptual comparison of family-based treatment and enhanced cognitive behavior therapy in the treatment of adolescents with eating disorders

**DOI:** 10.1186/s40337-019-0275-x

**Published:** 2019-12-31

**Authors:** Riccardo Dalle Grave, Sarah Eckhardt, Simona Calugi, Daniel Le Grange

**Affiliations:** 1 Department of Eating and Weight Disorders, Villa Garda Hospital, Via Montebaldo 89 1-37016 Garda (VR), Verona, Italy; 2 Center for the Treatment of Eating Disorders, Children’s Minnesota, Minneapolis, MN USA; 3 Department of Psychiatry, University of California, San Francisco, CA USA; 4 Department of Psychiatry and Behavioral Neuroscience, The University of Chicago, Chicago, IL USA

**Keywords:** Eating disorders, Anorexia nervosa, Treatment, Family-based treatment, Enhanced cognitive behavior therapy

## Abstract

**Background:**

The aim of this paper is to give a conceptual comparison of family-based treatment (FBT), a specific form of family therapy, and enhanced cognitive behavior therapy (CBT-E) in the management of adolescents with eating disorders.

**Main text:**

FBT and CBT-E differ in the conceptualization of eating disorders, the nature of involvement of parents and the child/adolescent, the number of treatment team members involved, and evidence of efficacy. FBT is the leading recommended empirically- supported intervention for adolescents with eating disorders. Data from randomized controlled trials indicate that FBT works well with less than half of the parents and adolescents who accept the treatment, but cannot be used with those who do not have available parents, or for those with parents who are not accepting of a FBT model, or are unable to participate in a course of this treatment. CBT-E has shown promising results in cohort studies of patients between ages 11 and 19 years, and has recently been recommended for youth with eating disorders when FBT is unacceptable, contraindicated, or ineffective.

**Conclusion:**

There is a need to compare these two treatments in a randomized controlled trial to assess their acceptability, effectiveness, relative cost and cost-effectiveness, and to explore moderators of treatment response.

## Plain English summary

Family-based treatment (FBT) is the current leading empirically-supported intervention for adolescents with eating disorders. As this treatment has certain limitations, alternative approaches are needed. The National Institute for Health and Care Excellence (NICE) has recently recommended cognitive behavior therapy (CBT) for eating disorders in children and young people when family therapy is unacceptable, contraindicated, or ineffective. This recommendation was supported by promising results demonstrated by the enhanced version of CBT (CBT-E), adapted for adolescents with eating disorders.

Given the importance of the NICE recommendation, this paper gives a brief overview of FBT and CBT-E, describes the main conceptual differences between these two treatments, and emphasizes the need to compare these two treatments in a randomized controlled trial to assess their acceptability, effectiveness, relative cost and cost-effectiveness, and to explore moderators of treatment response.

## Background

A specific form of family therapy, termed family-based treatment (FBT) [1], or at times referred to as the Maudsley method/Maudsley approach [2], is the current leading empirically-supported intervention for adolescents with eating disorders. As this treatment has certain limitations, alternative approaches are needed. The National Institute for Health and Care Excellence (NICE) has recently recommended cognitive behavior therapy (CBT) for eating disorders in children and young people when family therapy is unacceptable, contraindicated, or ineffective [3]. This recommendation was supported by promising results demonstrated by the enhanced version of CBT (CBT-E), adapted for adolescents with eating disorders [4, 5] in cohort studies of patients aged 11 to 19 years.

Given the importance of the NICE recommendation, the aim of this paper is to give a brief overview of FBT and CBT-E and a narrative review of the efficacy and effectiveness of the two treatments, and to describe the main conceptual differences between these two treatments.

### An overview of family-based treatment (FBT)

Family therapy for adolescent anorexia nervosa was originally developed in the late 1970s by a team of researchers at the Institute of Psychiatry and the Maudsley Hospital in London [2]. A behaviorally focused version of this original therapy has been described in detail [6], and has been manualized and named family-based treatment (FBT) [1]. In general, FBT does not align with a particular therapeutic approach, but instead integrates techniques from a variety of schools of psychotherapy, including systemic, strategic, narrative, and structural family therapy. The overall philosophy of FBT is that the adolescent with anorexia nervosa is embedded in the family, and that the parents’ involvement in the therapy is necessary for treatment success. Indeed, the overall perspective of FBT for adolescent anorexia nervosa is to see the family as a resource in the treatment of their child or adolescent [1].

FBT differs from other treatments of adolescent eating disorders for three main reasons [1]. First, the adolescent is not considered to be in control of his or her behavior, rather the treatment posits that the eating disorder controls the adolescent. The adolescent is seen as functioning as a much younger child in need of significant support from their parents. Second, the goal is to correct this position by improving the parents’ control over their adolescent’s eating. Frequently this control is abdicated, in part because parents either experience guilt for believing they have caused the adolescent’s eating disorder, or because the eating disorder symptoms have frightened them into inactivity or acting indecisively. Third, FBT focuses its effort on the task of weight restoration and to get the adolescent back onto a normal developmental trajectory, particularly in the first phase of the treatment, using an adaptation of the therapeutic family meal developed by the structural family therapy of Minuchin and his colleagues [7]. The primary goal is to keep parents focused on refeeding their adolescent, thus freeing the adolescent from the control of the eating disorder. This therapy is designed to consider adolescent developmental processes and return the adolescent to their developmental trajectory, though only after the patient has re-established a steady upward path.

A key aspect of the treatment is to separate the illness from the patient (i.e., to externalize the illness, or not identify the patient with the illness itself), in order to enable parents to take firm action against the eating disorder as opposed to acting against their daughter or son. FBT favors parents adopting an uncritical acceptance of the adolescent in their struggle against his or her symptoms. Parents are exonerated from blame for the patient’s illness and are congratulated on their earlier parenting skills. With few exceptions, parents are also encouraged to work out for themselves how to refeed their child with anorexia nervosa, and to view the therapist as a consultant who supports them in this effort. An important principle of FBT is therefore an agnostic view of the potential causes of anorexia nervosa, to help parents decrease guilt, and use their best resources to facilitate the patient’s recovery. The task of full parental engagement in treatment is achieved by appropriately raising their level of anxiety, by emphasizing the seriousness of the illness, which includes the risk of dying, and/or the difficulty of recovery.

Conjoint FBT involves the entire family (parents and siblings) attending each session, along with the unwell adolescent, although a version of this treatment has been delivered to parents alone while the adolescent meets with a nurse for no more than about 10 min at the start of each session (called parent-focused therapy (PFT)) and has been shown to be just as effective [8]. Whether delivered in conjoint or separated format, FBT typically includes no more than 20 sessions, each 50–60 min in length, with the exception of the second session, the family meal, which may for some families last up to 90 min.

**Fig. 1:**
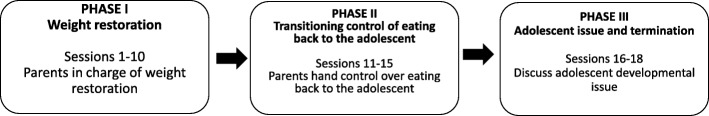
The three phases of family-based treatment

There are three phases in FBT (see Fig. 1). In Phase I, usually lasting about 3–4 months with sessions at weekly intervals, parents are charged with the responsibility of correcting their adolescent’s disordered eating behaviors and low weight. The therapist’s principal task is to assist the parents in developing and refining their strategies around this process. Once eating disorder behaviors are significantly reduced, control over food consumption is transferred back to the adolescent in an age appropriate fashion (in Phase II), and the sessions are gradually reduced from weekly to every second or even third week. In Phase III, once normal body weight (i.e., 95% median body mass index [BMI]) and eating behavior have been achieved, more general issues of adolescent development are addressed, and sessions are scheduled every third week or even at monthly intervals. A main theme in the last phase of this treatment is the creation of a healthy adolescent-parent relationship, which no longer requires the eating disorder as the basis of interaction. This requires increasing the adolescent’s autonomy, establishing appropriate intergenerational family boundaries, and helping the parents to recognize the need to reorganize their lives given their child’s pending departure from the family home [1].

The treatment team includes a primary clinician (e.g., child and adolescent psychiatrist, psychologist or social worker/family therapist), and a consulting team that could consists of a pediatrician, nurse, and dietitian [1], who meets with parents/patient as needed. Efforts should be made to concentrate the treating team within the same facility whenever possible, as close communication between providers is critical to the treatment’s success. For this reason, FBT may best be viewed as a complex and coordinated intervention, and although the most prominent component of the treatment is the focused psychotherapeutic intervention in terms of weight restoration, several sessions with a physician with extensive experience in medical treatment of adolescents with anorexia nervosa should be a key and indispensable component of this treatment. This is especially pertinent should the adolescent show signs of vital sign instability and a period of hospitalization, albeit briefly, should be required [9]. Similarly, a dietitian can be involved to help families with home-based refeeding, and to facilitate greater energy density and food variety [10].

### The efficacy of FBT for adolescents

The efficacy of manualized FBT for anorexia nervosa in adolescents has been tested in six randomized controlled trials (RCTs). Findings from these studies demonstrate an average remission rate, when this is defined as percent median BMI > 94% of expected for age, height, and gender, *and* an eating disorder examination score [11] within one standard deviation of population means, as < 40% for all participants at the end of treatment. On the other hand, treatment response, when this is broadly defined as an improvement in weight and eating-related psychopathology, averages near 75% [12]. Of note, no more than 15% of patients participating in FBT are typically hospitalized for acute medical instability for a mean duration of 7–10 days, before returning to the outpatient service for ongoing FBT [13]. Only one of the RCTs utilizing manualized FBT compared this therapy to an active individual comparison treatment, i.e., adolescent focused psychotherapy. On average, FBT is not significantly superior to individual adolescent treatment at post-treatment, but it does achieve greater symptom reduction by 6- and 12-month post-treatment follow-up [12]. For the most, the remaining RCTs utilizing FBT have compare it to different forms of family engagement in treatment. At this time there are no studies which have compared FBT to CBT-E.

FBT for bulimia nervosa has been compared across two RCTs; FBT-BN vs. individual supportive psychotherapy [14], and FBT-BN vs. CBT adapted for adolescents (CBT-A), a treatment derived from the CBT for bulimia nervosa [15, 16]. In the 2007 study, participants in FBT-BN, remitted at significantly higher rates at end of treatment (39% versus 18%), and at 6-month follow-up (29% versus 10%). For the 2015 study, participants in FBT-BN achieved significantly higher abstinence rates than in CBT-A at end of treatment (39% versus 20%), and at 6-month follow-up (44% versus 25%), but abstinence rates between these two groups were no longer statistically significant at 12-month follow-up (49% versus 32%).

### An overview of enhanced cognitive behavior therapy

CBT-E is an evidence-based treatment that has been developed for addressing the psychopathology of all eating disorders, as opposed to the specific diagnoses outlined in the Diagnostic and Statistical Manual of Mental Disorders (DSM-5) [17]. Although originally designed for adults, it has now been adapted for adolescents with eating disorders [4, 5].

Whereas FBT is based on the concept that the problem or symptoms belong to the entire family [1], CBT-E views the problem as belonging to the individual. CBT-E is therefore designed to treat the eating disorder as part of the patient, and encourages the patient, not their parents, to take control. CBT-E treatment procedures involve patients actively in all phases of treatment, with the aim of promoting a feeling of self-control; it is the patient that has the final say, not only in the decision to start treatment, but also which problems to address, and which procedures will be used to address them.

CBT-E is a collaborative approach to overcoming problems with eating (collaborative empiricism), whatever they may be. Patients are encouraged to actively participate in the process of change, and to consider the treatment a priority. The CBT-E therapist always keeps their patients fully apprised of what is happening, informing them that it will not be easy, but it will be worthwhile to take steps to overcome their eating problem. In order to avoid increasing any resistance to change, there are no “coercive” or “prescriptive” procedures involved in CBT-E; patients are never asked to do things that they do not agree to. Indeed, one of the four major goals of CBT-E for adolescents is to engage them in the treatment, involving them actively in the process of change.

The second major goal of CBT-E is to deal with the eating disorder psychopathology. This will involve addressing patients’ concerns about shape, weight and eating, as well as any dietary restraint and restriction (and low weight if applicable), and extreme weight control behaviors. As part of this process, patients are encouraged to understand and disrupt the mechanisms maintaining their eating disorder psychopathology—the third major treatment goal—which are illustrated to them through the collaborative creation of a personal formulation. This is a key strategy, as it highlights the targets of the treatment to come, and thereby helps guide a made-to-measure approach for addressing the evolving psychopathology of each individual patient. As part of this approach, patients are educated about the processes that characterize their personal formulation, which can be modified mid-course to address any emerging processes, and actively involved in the decision to tackle them. This promotes self-empowerment, and helps them to conclude that they have a problem that needs addressing.

Once the patient has reached this conclusion, which is an essential prerequisite of such a collaborative form of treatment, they are gradually introduced to a flexible set of sequential cognitive and behavioral strategies and procedures, as well as further education, designed to progressively address their personal eating disorder psychopathology and its maintenance mechanisms. These strategies will need to be practiced at home, and it is what the patient does between sessions that will determine the treatment outcome. To achieve cognitive change, patients are encouraged to observe how the processes in their personal formulation operate in real life. This is achieved through real-time self-monitoring. Strategically planned homework tasks, making gradual behavioral changes and analyzing their effects and implications on their way of thinking, are also central to the treatment, but need to be integrated with care, as they may provoke anxiety. To keep the patient on track, the therapist therefore needs to be both empathetic and firm about what needs to be achieved.

The fourth major goal of CBT-E is for the patient to achieve lasting change. Hence, in the later stages of CBT-E, the treatment shifts to a future-oriented approach. When the main maintenance processes outlined in the patient’s personal formulation have been disrupted, and they experience periods of no concerns about their shape, weight, and eating, they can be educated about their eating disorder mindset, and helped to become aware of the signs that this is reactivating. They can then be provided with strategies designed to help them decenter from it quickly, and thereby ward off relapse [18].

**Fig. 2:**
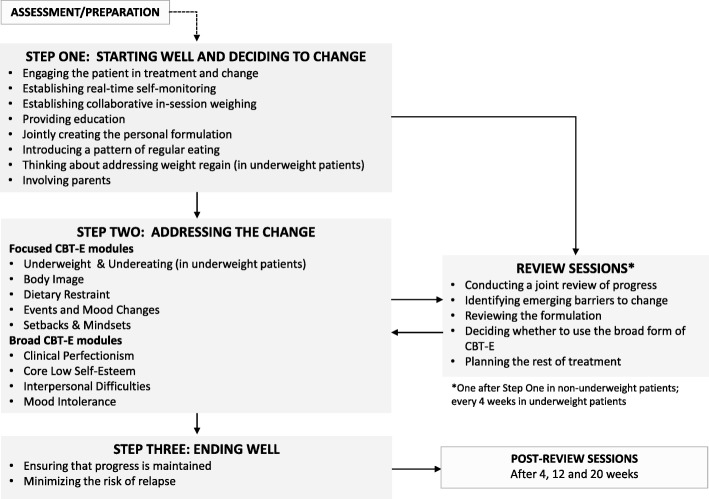
The enhanced cognitive behavior therapy (CBT-E) map for adolescents with eating disorders

CBT-E for adolescents starts with two sessions designed to assess the patient’s needs and prepare them for treatment. The treatment is then delivered by a single therapist in three main Steps (see Fig. 2), each with a different emphasis. The first is geared towards patients’ reassessing their current state, and how what they do affects them. Patients are then asked to consider the pros and cons of tackling their low weight (if applicable). In the second Step, patients (if willing) are provided with the tools they will need to address their eating-disorder psychopathology by alleviating concerns about shape and weight, and assisted with weight restoration (if necessary). In the final Step of CBT-E, the emphasis is on helping patients to develop personalized strategies for rapid recovery from setbacks, and thereby to maintain the changes that they have achieved in the long term.

In patients with a BMI between the 3rd and 25th centile, treatment is generally delivered over the course of 30–40, 50-min sessions, whereas those with a BMI > the 25th centile attend 20 such sessions. As in CBT-E for adults [18], however, the treatment duration is flexible, as it will depend on the items that need to be addressed. Hence, in a review session held after 4 weeks in non-underweight patients, or in one of the review sessions in Step 2 in underweight patients, the decision is taken to use either the “focused” form, which addresses only the specific features of eating-disorder psychopathology, or the “broad” form, which is designed to address any “external” mechanisms, i.e., clinical perfectionism, core low self-esteem, mood intolerance, and/or interpersonal difficulties, that may be operating. These are tackled using specific, additional CBT-E modules, and will therefore require the treatment to be extended. In most patients, the focused form is appropriate, but the broad form should be considered if in the review sessions it is concluded that one or more of the external mechanisms maintaining the eating disorder psychopathology [4]: (i) are pronounced; (ii) appear to be maintaining the eating disorder; and (iii) seem likely to interfere with the response to treatment.

Naturally, parents are not excluded from participating in their child’s treatment, but their involvement is limited to helping create a family environment conducive to recovery. To this end, during the first two weeks of treatment, they are invited to attend a single one-hour assessment session, held immediately after an individual session with the patient, with the aim of identifying family-related factors capable of undermining their child’s efforts to change. This session is held with the parents alone, but is followed by subsequent sessions (four to six times in not-underweight patients, eight to twelve in those who present as underweight) each lasting 15–20 min, with the patient and parents together at the end of a patient’s individual session. In general, a date is set for the first joint parent-patient session after the introduction of the regular eating procedure, both in underweight and not underweight patients; this session should be dedicated to explaining how parents may help the patient to implement it. Other joint sessions may be set up when the underweight patient has made the decision to address weight restoration, in order to discuss the parents’ role before, during, and after meals. Finally, it may be helpful to involve parents in order to help the patient implement some procedures of the CBT-E broad modules, both in underweight and not underweight patients. During these sessions, parents are kept abreast of how treatment and their child are progressing, and anything they can do to help. In order to promote a sense of self-determination, anything that will be discussed in these sessions is negotiated with and agreed to by the patient beforehand.

### The effectiveness of CBT-E for adolescents

To date, four different cohort studies, on patients aged between 11 and 19 years, have been conducted to assess outpatient CBT-E for adolescents. Findings from these studies showed that in patients with anorexia nervosa who complete the treatment (60–65%) about 60% achieved a full response (i.e., BMI centile corresponding to an adult BMI of ≥18.5 kg/m^2^ and an eating disorder examination interview score [11] within one standard deviation of population means).

Three of the four studies investigated the effects on patients with anorexia nervosa, and one on non-underweight adolescents with other eating disorders. In the first study, 49 adolescents with anorexia nervosa were given 40 outpatient CBT-E sessions, and a significant increase in BMI-for-age percentile, from 3.36 (SD = 3.73) to 30.3 (SD = 16.7), along with a marked improvement in eating-disorder psychopathology and general psychiatric features, was seen in two-thirds of completers [19]. At 60-week follow-up, these positive outcomes remained almost unchanged despite minimal subsequent treatment. These encouraging findings were mirrored by a subsequent study of outpatient CBT-E involving 68 non-underweight adolescents with an eating disorder [20], three-quarters of whom completed the full 20-session program. Intent-to-treat analysis revealed that, at the end of treatment, 68% of patients displayed only minimal residual eating-disorder psychopathology, and half of those with prior episodes of binge-eating or purging reported no longer having them.

Interestingly, a comparative study of CBT-E efficacy in 46 adolescents and 49 adults with anorexia nervosa [21] revealed that weight normalization occurred in considerably more adolescents than adults (65.3% vs. 36.5%). Furthermore, weight restoration was achieved roughly 15 weeks earlier on average by adolescents vs. adults. These findings provide compelling evidence not only that CBT-E is even more effective in adolescent patients, but also that positive outcomes may be achieved in a shorter time frame than that required by adults.

Finally, a more recent study set out to assess the outcomes and determine the predictors of change in a cohort of 49 adolescent patients with marked anorexia nervosa, treated with outpatient CBT-E in a real-world setting [22]. More than 95% of patients accepted the treatment and 71.4% completed it, displaying a large increase in weight, together with a marked decrease in eating-disorder and general psychopathology, and clinical impairment scores. These changes were maintained at six-month follow-up, suggesting that CBT-E is a promising treatment for adolescents with anorexia nervosa when it is also delivered in a real-world setting, even though no baseline predictors of drop-out and treatment outcome were found. The percentage of drop-out was higher than those reported in most recent FBT studies (15–20%) [13], but this difference could be in part explained by the criteria used to define drop-out - e.g., 15% of the patients hospitalized during the course of the treatment were included as completers in the FBT study [13], whereas all the hospitalized patients (8.2%) were considered drop-outs in the present study. It also underlines that BMI percentile for age and gender at end of treatment and 20-week follow-up was broadly similar to that reported in the recent FBT studies.

### Major differences between FBT and CBT-E

FBT and CBT-E differ in the conceptualization of eating disorders, the nature of involvement of parents and child/adolescent, the number of treatment team members involved, and evidence of efficacy (see Table 1).

**Table 1:** Principal differences between family-based treatment (FBT) and enhanced cognitive behavior therapy (CBT-E)

	FBT	CBT-E
Conceptualization of eating disorders	The problem belongs to the entire family The illness is separated from the patient	The problem belongs to the individual It does not separate the illness from the patient
Adolescent’s involvement	Not actively involved	Actively involved
Parents’ involvement	Vitally important	Useful but not essential
Treatment team	Multidisciplinary	Single therapist
Sessions (n)	18 family sessions Sessions with the consulting team (paediatrician or nurse) in case of need for hospitalization (~ 15%)	20 individual sessions (non-underweight patients) 30–40 individual sessions (underweight patients)

### Conceptualization of eating disorders

In FBT, the problem or symptoms belong to the entire family, and therapy works to separate the illness from the patient (externalization), enabling parents to temporarily take control of their child or adolescent’s eating. Several schools of thoughts contributed to the type and style of this treatment. The family meal, for example, is derived from Structural Family Therapy [7], which postulates that the child is physiologically vulnerable, and has a critical role in the family’s avoidance of conflicts that acts as a powerful reinforcement of symptoms. Through family meals parents reinforce the effectiveness of their own parental dyad and the adolescent’s emotional involvement with her/his parents is reduced. The strategy of maintaining an agnostic view regarding the causes of anorexia nervosa is derived from Strategic Family Therapy [23, 24], and has the aim of limiting the impact of the symptoms on the patient and family, and to focus the therapy on the problematic patterns that are maintaining the eating disorder. Additionally, the strategy of encouraging the parents to find solutions that work for them, rather than relying on the outside authority of the therapist, while the therapist holds the family and their efforts in a positive and noncritical way, are derived from the Milan Systems Therapy [25]. This school of thought postulates that the family is a rigid organized homeostatic mechanism, resistant to change from the outside. Separating the illness from the adolescent, or externalization, comes from Narrative Therapy [26], and finally, feminist theory, emphasizing the need for partnership and shared control of the therapeutic process [23], has been used to form a sincere partnership between the therapist and the parents with the healthy part of the adolescent’s growth process, even if it defiles parental will.

The initial focus of FBT is the task of weight restoration through the parents’ efforts at home. Once this is achieved the focus gradually shifts toward adolescent issues with the family, and the therapist encourages the family to examine the relationship between adolescent issues, working towards increased personal autonomy for the adolescent, and establishing more appropriate intergenerational boundaries. Toward the end of treatment, the therapist will check with the parents, if appropriate for the age and developmental stage of the children, regarding their need to reorganize their life together after the child’s prospective departure from the family home [1]. FBT does not directly address the underlying theoretical constructs of CBT-E, such as overvaluation of shape and weight, event and moods influencing eating, and external clinical features (e.g., clinical perfectionism, core low self-esteem, mood intolerance, interpersonal problems), although it strongly encourages peer social interaction.

CBT-E, on the other hand, views the illness as belonging to the individual. Cognitive behavioral theory postulates that these patients have a shared but distinctive self-evaluation scheme based on their overvaluation of shape and weight which plays a central role in maintaining all eating disorders [17]. This “core psychopathology” gives rise, directly or indirectly, to the other clinical features of the disorder, whatever its DSM-5 classification. These clinical features are therefore explored with the patient and laid down in an evolving personal formulation. These clinical “expressions” of the patient’s eating disorder and the mechanisms that act to reinforce them are then targeted by a progressive series of well-specified CBT strategies and procedures designed to help patients to change their behavior and reflect on the consequences of these changes. The ultimate aim is to train patients how to de-centre from and overcome their difficulties, and, thus, for them to learn to control their eating-disorder mindset, rather than the mindset controlling them [18].

### Involvement of parents and adolescent

Parents’ involvement in FBT is vitally important for the ultimate success of the treatment. Moreover, in FBT, parents must defer working on other family conflicts or disagreement until the eating-disorder behaviors are resolved. Parents’ involvement in CBT-E is useful but not essential. The role of parents, as described above, is only simply to support the implementation of the one-to-one treatment. Both treatments pay attention to adolescent development, however, in FBT the adolescent is not viewed as being in control of his/her behavior (the eating disorder controls the adolescent), and this is corrected by improving the parental control over eating in the first phase of the treatment. On the contrary, in CBT-E the adolescent is helped to learn how to control his/her behavior, and parents may be involved in helping the adolescent in pursuing this task. In FBT the adolescent is initially not actively involved, and plays a more passive role, although his/her role becomes more active in the last phase of the treatment, while in CBT-E the adolescent is encouraged form the beginning to become actively involved in the treatment.

### Treatment team

FBT is made up of a variety of key components, each of which may contribute to its effects. Most prominent is the psychotherapeutic element, focusing on weight restoration, and is delivered by a primary clinician (e.g., child and adolescent psychiatric, psychologist or social worker/family therapist). In the most recent FBT trials, this involved no more than 20 one-hour family sessions over about nine months [1]. Another component are the sessions with a physician with expertise in the medical management of adolescents with anorexia nervosa. These meetings usually start out weekly, before tapering off to monthly or six-weekly, as is clinically indicated. Hospitalization for medical instability should be pursued when indicated.

CBT-E is provided by a one therapist (e.g., psychologist or a health professional trained in the treatment) who is substituted when they have to be absent. It is delivered in 20 treatment sessions over 20 weeks (in not underweight patients) and 30–40 sessions over 30–40 weeks (in underweight patients). The treatment also involves a 90-min assessment session with only the parents and some 15–20 min sessions with the patient and parents together (see above). No additional therapeutic input, either from physicians, dieticians, or other health professionals, other than an initial assessment by a physician to check that the patient is suitable for outpatient treatment and reassessment if there were physical concerns (e.g., due to weight loss or frequent purging), is required. Patients who are hospitalized are not included in the outcome as they are considered non-responders to the treatment.

### Similarities between FBT and CBT-E

Despite several differences, the general strategy of FBT and CBT-E is to address the maintaining mechanism of the eating disorder psychopathology, as opposed to an exploration of any potential causes of the eating disorder psychopathology. Indeed, both treatments take an agnostic view of the cause of the illness (i.e., no assumptions are made about the potential origins of eating disorders).

A major focus of both treatments is to help the adolescent patient to normalize body weight and to support the adolescent’s return to a normal developmental trajectory of weight. Both FBT and CBT-E, although using different procedures, include regular weighing of the patients within each session. The focus of both FBT and CBT-E in addressing dietary restriction and low weight has led to suggest that perhaps one common mechanism of action of the two treatments might be exposure (and habituation) to feared food and its consumption [27].

Another possible mechanism of action shared by FBT and CBT-E is how they might indirectly reduce the over-evaluation of shape and weight once the patient has normalized weight: CBT-E helps the patient to enhance the importance of other domains of life (e.g., school, social life, hobbies, etc.), while FBT works toward increased personal autonomy for the adolescent.

Finally, both FBT and CBT-E set out to manage comorbid psychiatric diagnoses by involving a psychiatrist as part of the care team. Hospitalization, for psychiatric or medical acuity, is recommended only when the patients present with clinical severity that cannot or should not be managed in an outpatient setting.

## Conclusions

FBT is the current evidence-based treatment for eating disorders in adolescents, as its efficacy has been assessed by several RCTs. However, the treatment presents a number of challenges. First, there are no direct comparisons of FBT with CBT-E or with other psychological treatments combined with nutritional rehabilitation aimed at weight restoration. Second, the current research evidence suggests that FBT works well with about two third of the parents and adolescents who accept the treatment, although less than 40% achieve a full remission. However, it cannot be used with those adolescents who do not have available parents, or for those with parents who are not accepting of a family-based treatment model. Third, even among those who do accept the treatment, there are sometimes difficulties implementing FBT given the expectation that all members of the family be actively involved, which may necessitate parents taking time away from work, disrupting sibling’s schedules and creating complicated travel arrangements.

CBT-E is recommended for adolescents when FBT is unacceptable, contraindicated, or ineffective [3]. This recommendation is based on the promising findings derived by some cohort studies, and it is reinforced by a recent study in a real-world setting, showing outcome data similar to those reported by FBT [22].

The availability of two effective treatments for adolescents with eating disorders now opens the chance to compare them in a randomized controlled trial. Key variables of interest would include the acceptability of the two treatments, their short- and long-term efficacy, their cost cost-effectiveness, and the treatment response moderators that might allow the matching of adolescent patients to CBT-E or FBT. This is not improbable as they differ markedly in their strategies, procedures and postulated mechanisms of action. The atheoretical nature of the FBT [28] might suggest that it is well suited to the needs of younger adolescent patients. In contrast, CBT-E might have its greatest effect in older adolescent patients in whom the mechanisms that maintain eating disorder psychopathology are fully operating [17]. Older adolescents might also better accept an “adult” form of treatment rather than a family style one. However, these hypotheses require testing through a future RCT.

## Data Availability

Not applicable.
